# *Staphylococcus aureus* Protein A Mediates Interspecies Interactions at the Cell Surface of *Pseudomonas aeruginosa*

**DOI:** 10.1128/mBio.00538-16

**Published:** 2016-05-24

**Authors:** Catherine R. Armbruster, Daniel J. Wolter, Meenu Mishra, Hillary S. Hayden, Matthew C. Radey, Gennifer Merrihew, Michael J. MacCoss, Jane Burns, Daniel J. Wozniak, Matthew R. Parsek, Lucas R. Hoffman

**Affiliations:** a Department of Microbiology, University of Washington, Seattle, Washington, USA; b Department of Pediatrics, University of Washington, Seattle, Washington, USA; c Department of Genome Sciences, University of Washington, Seattle, Washington, USA; d Department of Microbial Infection and Immunity, The Ohio State University, Columbus, Ohio, USA

## Abstract

While considerable research has focused on the properties of individual bacteria, relatively little is known about how microbial interspecies interactions alter bacterial behaviors and pathogenesis. *Staphylococcus aureus* frequently coinfects with other pathogens in a range of different infectious diseases. For example, coinfection by *S. aureus* with *Pseudomonas aeruginosa* occurs commonly in people with cystic fibrosis and is associated with higher lung disease morbidity and mortality. *S. aureus* secretes numerous exoproducts that are known to interact with host tissues, influencing inflammatory responses. The abundantly secreted *S. aureus* staphylococcal protein A (SpA) binds a range of human glycoproteins, immunoglobulins, and other molecules, with diverse effects on the host, including inhibition of phagocytosis of *S. aureus* cells. However, the potential effects of SpA and other *S. aureus* exoproducts on coinfecting bacteria have not been explored. Here, we show that *S. aureus*-secreted products, including SpA, significantly alter two behaviors associated with persistent infection. We found that SpA inhibited biofilm formation by specific *P. aeruginosa* clinical isolates, and it also inhibited phagocytosis by neutrophils of all isolates tested. Our results indicate that these effects were mediated by binding to at least two *P. aeruginosa* cell surface structures—type IV pili and the exopolysaccharide Psl—that confer attachment to surfaces and to other bacterial cells. Thus, we found that the role of a well-studied *S. aureus* exoproduct, SpA, extends well beyond interactions with the host immune system. Secreted SpA alters multiple persistence-associated behaviors of another common microbial community member, likely influencing cocolonization and coinfection with other microbes.

## IMPORTANCE

Bacteria rarely exist in isolation, whether on human tissues or in the environment, and they frequently coinfect with other microbes. However, relatively little is known about how microbial interspecies interactions alter bacterial behaviors and pathogenesis. We identified a novel interaction between two bacterial species that frequently infect together—*Staphylococcus aureus* and *Pseudomonas aeruginosa*. We show that the *S. aureus*-secreted protein staphylococcal protein A (SpA), which is well-known for interacting with host targets, also binds to specific *P. aeruginosa* cell surface molecules and alters two persistence-associated *P. aeruginosa* behaviors: biofilm formation and uptake by host immune cells. Because *S. aureus* frequently precedes *P. aeruginosa* in chronic infections, these findings reveal how microbial community interactions can impact persistence and host interactions during coinfections.

## INTRODUCTION

The majority of research on bacterial infections has focused on individual species. However, many diseases are caused by consortia of coinfecting microbes. For example, *Staphylococcus aureus* is an opportunistic pathogen that frequently infects along with other bacteria in a range of diseases (1, 2). Many of the most common and devastating of these infections afflict the heart, blood vessels, and lungs (3, 4) (particularly those of transplant and cystic fibrosis [CF] patients [5]), as well as those of skin wounds, medical devices, and the urinary tract (6, 7). These infections frequently result in chronic persistence (8) and dissemination (9), two disease characteristics that are often attributed to the formation of biofilms: dense aggregates of bacteria encased in a protective extracellular matrix (10). Biofilm matrix composition differs among species, but it generally includes exopolysaccharides (EPS), proteins, and extracellular DNA (eDNA) (11). While biofilm matrices have traditionally been thought to play a primarily passive, structural role, recent studies have identified additional matrix functions, such as selective retainment of bioactive proteins (12) and even acting as signaling molecules (13).

The airways of CF patient lungs are host to chronic infections that are typically polymicrobial. While *S. aureus* is the bacterium cultured most frequently from CF patient sputum samples, *Pseudomonas aeruginosa* increases in prevalence as patients age (14). Coinfection with *P. aeruginosa* and *S. aureus* occurs in approximately 40% of U.S. children with CF (15) and has been associated with increased airway inflammation, reduced lung function, and increased mortality (16, 17). These infections are notoriously persistent despite aggressive antibiotic treatment and robust host inflammation, and evidence indicates that biofilm formation contributes to this recalcitrance (18–20).

Studies have found that *P. aeruginosa* and *S. aureus* can alter the behavior of each other *in vitro*, suggesting that coinfection could impact each species’ pathogenesis and persistence (21–25). Most of the research on these interactions has focused on how *P. aeruginosa* influences *S. aureus* (21, 23, 24, 26). For example, *P. aeruginosa*-secreted products can inhibit the growth of *S. aureus*, influencing its response to antibiotics (21). *P. aeruginosa* exoproducts can lyse *S. aureus* and extract its iron stores for growth (22), as well as induce airway epithelial cells to kill *S. aureus* and other Gram-positive bacteria (27). Therefore, many of the known interactions between these two species are mediated by *P. aeruginosa* exoproducts. By comparison, the impact of *S. aureus* on *P. aeruginosa* behavior is less understood. As *S. aureus* usually infects at high densities when *P. aeruginosa* is acquired during CF and other conditions (14), studying the impact of *S. aureus* on *P. aeruginosa* behaviors has the potential to reveal whether *S. aureus* influences downstream infection by *P. aeruginosa*.

*S. aureus* secretes a large repertoire of exoproducts that could affect other bacteria and the host. This list includes 21 different extracellular adhesins (which bind a variety of host targets), a large variety of enzymes (including proteases, autolysins, and nucleases) and small molecules (such as pore-forming exotoxins and surfactant peptides), extracellular DNA, and a polysaccharide (28). Together, these *S. aureus* molecules can mediate attachment to and invasion of eukaryotic cell surfaces, as well as evasion of the host immune response, leading to persistent and recurring infections. However, how these factors impact the microbes that frequently coinfect with *S. aureus* has not been explored.

The goal of this study was to evaluate the potential of *S. aureus* extracellular products to influence *P. aeruginosa* behaviors relevant for persistent infection, including biofilm formation and survival against the host immune response. To identify *S. aureus* products that interact with *P. aeruginosa*, we first used a high-throughput biofilm assay to screen a collection of *P. aeruginosa* CF clinical isolates for alteration of biofilm formation when exposed to *S. aureus* cell-free culture supernatant. Here we report the first evidence of a role for a well-studied *S. aureus* adhesin, staphylococcal protein A (SpA), in mediating bacterial interspecies interactions and for altering the interaction of another bacterial species with host immune cells. We found that SpA exerts these effects by binding to *P. aeruginosa* cell surface targets. These findings highlight the importance of considering the role of bacterial exoproducts, which have been traditionally studied in pure culture, in multispecies infections.

## RESULTS

### *S. aureus* alters *P. aeruginosa* biofilm formation.

**FIG 1:**
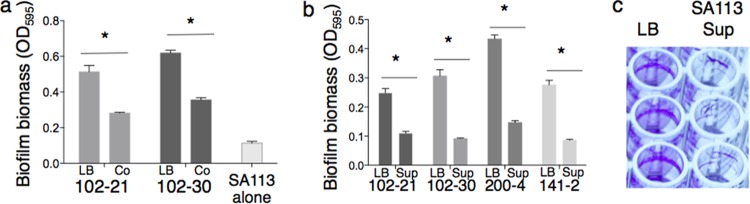
(a) Clonally related *P. aeruginosa* isolates from patient 102 exhibited inhibition of biofilm formation when grown in coculture with *S. aureus* SA113, compared to monoculture in LB. All experiments shown are from crystal violet-stained biofilms after 4 h of growth. The pairs of bars in the graph represent results for *P. aeruginosa* cells grown in monoculture in LB (left) or results for coculture with *S. aureus* S113 (right). (b) *P. aeruginosa* respiratory isolates from multiple CF patients, including patient 102, displayed biofilm inhibition when grown in *S. aureus* SA113 supernatant, compared with growth in LB. Images are from experiments using crystal violet-stained biofilms after 4 h of growth. Each pair of bars in the graph represent results for growth in LB (left) and growth in SA113 supernatant (right). For panels a and b, an asterisk indicates that the biofilm biomass of patient 102 *P. aeruginosa* isolates grown in SA113 supernatant differed significantly from that of each strain grown in LB (Student’s *t* test; *P* < 0.05). (c) A representative image of the biofilm inhibition phenotype in the crystal violet assay. The isolate shown is *P. aeruginosa* 102-21, after 4 h of growth in buffered medium (LB-MOPS) or cell-free SA113 supernatant after growth in the same medium.

In order to identify interspecies interactions relevant to establishing a chronic infection, we initially screened a small collection of *P. aeruginosa* respiratory isolates from children with CF for altered biofilm formation during coculture with the lab strain *S. aureus* SA113 (29) and also in SA113 cell-free culture supernatant (SA113 Sup). We observed that coculturing with *S. aureus* resulted in a significant decrease in biofilm biomass formed by many of the *P. aeruginosa* clinical isolates (9 of 24 isolates tested) (see Table S2 in the supplemental material) compared to that formed by these isolates when grown in pure culture (Fig. 1a). Plating experiments showed the resulting biofilms to be comprised almost entirely of *P. aeruginosa* (data not shown). Furthermore, culture supernatant derived from *S. aureus* SA113 inhibited *P. aeruginosa* biofilm formation on plastic surfaces as well as coculture did, indicating that biofilm inhibition activity was attributable to a molecule released by *S. aureus* into the medium (Fig. 1b and c). Notably, inhibited isolates still formed air-liquid interface aggregates (pellicles) in SA113 supernatant, suggesting that the observed biofilm inhibition was due to decreased attachment to abiotic surfaces, but not decreased cell-cell adhesion. As an aside, we also observed that biofilm formation was stimulated, rather than inhibited, by *S. aureus* culture supernatant in 15 of 24 isolates tested (see Table S2). We have found this biofilm-stimulatory phenotype to be attributable to an *S. aureus*-secreted molecule that is different from the biofilm-inhibitory *S. aureus* product described here; this stimulatory activity will be the subject of a forthcoming manuscript.

To determine whether other *S. aureus* strains were able to inhibit *P. aeruginosa* biofilm formation, we measured biofilm formation by *P. aeruginosa* clinical isolate 102-21 in cell-free supernatants from two additional *S. aureus* laboratory strains and from 7 CF *S. aureus* isolates from the same children from whom the *P. aeruginosa* isolates were collected (including two isolates from patient 102, the source of the test *P. aeruginosa* isolate). All supernatants tested had the same inhibitory activity as SA113 (see Table S3 in the supplemental material).

### *S. aureus* secretes a protein(s) responsible for biofilm inhibition.

To identify the molecule(s) in *S. aureus* supernatant responsible for *P. aeruginosa* biofilm inhibition, we first subjected SA113 Sup to various physical and chemical treatments in an attempt to abrogate the inhibition of biofilm formation by *P. aeruginosa* 102-21. We found boiling and proteinase K treatment eliminated biofilm inhibition by SA113 Sup (see Fig. S1 in the supplemental material). Centrifugation with molecular-weight-cutoff filters indicated that the molecule(s) conferring biofilm inhibition activity had a mass larger than 30 kDa (see Fig. S1). Biofilm inhibition activity was not impacted by treatment of the supernatant with either DNase I or RNase (see Fig. S1). These data suggest that biofilm inhibition is attributable to at least one protein larger than 30 kDa.

### The secreted protein SpA is responsible for *P. aeruginosa* biofilm inhibition.

In order to identify the protein(s) responsible for inhibiting biofilm formation, *S. aureus* SA113 supernatant was fractionated by size-exclusion chromatography, and the fractions were tested for biofilm-inhibitory activity. Liquid chromatography-tandem mass spectrometry (LC-MS/MS) was performed on the four fractions that inhibited *P. aeruginosa* biofilm formation (see Fig. S2 in the supplemental material), as well as on total supernatant and 3 inactive fractions. The normalized spectral abundance factor (NSAF) approach (30) was used to characterize the relative abundances of specific proteins in active fractions compared to inactive fractions. The NSAF for an *S. aureus* adhesin, SpA, was increased 10-fold in active versus inactive fractions (NSAF of 0.2 in active, 0.02 in control), identifying it as a candidate biofilm inhibitor. SpA is one of a variety of broadly adhesive extracellular proteins produced by *S. aureus* known as microbial surface components recognizing adhesive matrix molecules, or MSCRAMMs. While most studies of SpA have focused on the fraction that is covalently anchored to the cell wall, a significant amount of SpA is known to be released into the extracellular milieu during growth (31, 32).

**FIG 2:**
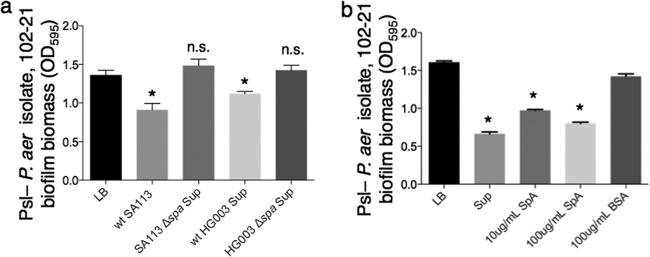
(a) Inhibition of biofilm formation by *P. aeruginosa* isolate 102-21 is lost in the absence of SpA. Culture supernatant from *S. aureus* lab strain HG003 exhibited biofilm inhibitory activity against isolate 102-21, but HG003 Δ*spa* supernatant did not. An asterisk indicates a significant difference in biofilm biomass compared to the LB control (*P* < 0.05); n.s., not statistically significant compared to the LB control (*P* > 0.05). (b) Purified SpA inhibited biofilm formation by Psl^−^ *P. aeruginosa* in a 4-h crystal violet assay*.* An asterisk indicates a significant difference in biofilm biomass compared to that of the LB control (*P* < 0.001).

To determine if SpA directly inhibits *P. aeruginosa* biofilms, we constructed a clean deletion of the *spa* gene in two *S. aureus* genetic backgrounds that produced inhibitory activity, SA113 and HG003. Biofilm inhibition of *P. aeruginosa* 102-21 by supernatants from these mutants was greatly reduced compared to inhibition by wild-type supernatants (Fig. 2a). Additionally, purified SpA directly added to unconditioned medium resulted in biofilm inhibition (Fig. 2b). It is possible that other MSCRAMMs produced by *S. aureus*, a few of which were identified in total SA113 Sup by LC-MS/MS but not enriched in active fractions (data not shown), could also inhibit *P. aeruginosa* biofilm formation. Thus, we tested five MSCRAMM transposon insertion mutants and their parent strain, *S. aureus* JE2. Of these, only mutants defective for SpA production significantly lost biofilm inhibition activity (see Fig. S3 in the supplemental material). Therefore, SpA was primarily responsible for biofilm inhibition by *S. aureus* supernatant. We performed semiquantitative Western blotting with an anti-SpA antibody on 6-h cell-free *S. aureus* SA113 culture supernatant in order to estimate the concentration of SpA present, which was approximately 100 µg/ml (data not shown).

### SpA binds to Psl and type IV pili on the *P. aeruginosa* cell surface.

We hypothesized that SpA inhibited *P. aeruginosa* biofilm formation by binding to specific targets on the cell surface required for surface adhesion. To test this hypothesis, we compared the abilities of representative *P. aeruginosa* isolates that did and did not exhibit biofilm inhibition (102-21 and 102-2, respectively) to bind fluorescently labeled SpA (fluorescein isothiocyanate [FITC]-SpA). We found that 102-2 bound an amount of FITC-SpA comparable to that bound by wild-type laboratory strain PAO1 (which also does not exhibit biofilm inhibition) and that each bound more SpA than 102-21 (see Fig. S4a in the supplemental material). Therefore, the isolate that exhibited biofilm inhibition (102-21) bound the least SpA of the three tested, suggesting that this isolate lacks one or more SpA binding targets present in uninhibited isolates.

**FIG 3:**
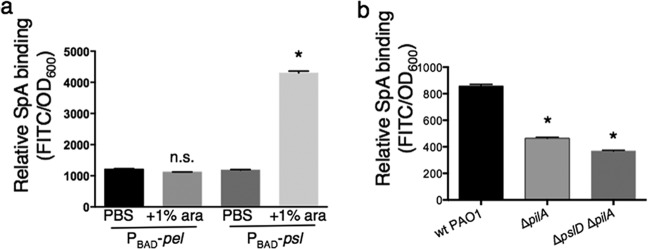
(a) SpA binds to *P. aeruginosa* Psl. Shown are fluorescence levels of FITC-SpA after incubation with the indicated strains, followed by washing. The asterisk indicates a significant difference in relative FITC fluorescence compared to the PBS control (*P* < 0.05); n.s., not statistically significant compared to the PBS control (*P* > 0.05). (b) FITC-SpA binding assay (also shown in Fig. S5a in the supplemental material) with the indicated strains, suggesting that SpA binds to *P. aeruginosa* surface type IV pili. Asterisks indicate a significant difference in relative FITC fluorescence compared to that of wild-type PAO1 (*P* < 0.05).

To identify the binding targets of FITC-SpA on *P. aeruginosa*, we screened a collection of PAO1 mutants and overexpression strains for relative SpA binding. *P. aeruginosa* produces at least three exopolysaccharides, two of which play a role in biofilm formation by nonmucoid strains (Pel and Psl [34]). We found that overexpression of Psl, but not Pel, exhibited increased FITC-SpA binding (Fig. 3a), suggesting that SpA bound to Psl. Similarly, a mutant known to overproduce Psl (MPAO1 Δ*wspF*) exhibited enhanced FITC-SpA binding relative to wild type, but a deletion of the *pslD* gene in the Δ*wspF* genetic background abrogated this effect (MPAO1 Δ*wspF* Δ*pslD*) (see Fig. S4b in the supplemental material). To further test whether SpA binds Psl, we performed coimmunoprecipitations with SpA-coated beads and *P. aeruginosa* culture supernatant followed by immunoblot assays with anti-Psl antibody, and we demonstrated SpA-Psl binding (see Fig. S5a in the supplemental material). To determine whether SpA bound to Psl directly or whether binding was indirect and required a third molecule, such as the known Psl binding adhesion factor CdrA, we repeated the SpA/Psl coimmunoprecipitation using boiled *P. aeruginosa* culture supernatant (see Fig. S5a, bottom row) and supernatant from MPAO1 Δ*cdrA*. We found that Psl continued to coimmunoprecipitate with SpA, suggesting that SpA does not require an additional Psl binding protein from *P. aeruginosa* in order to bind to Psl.

Additionally, we found that SpA bound a component of the type IV pilus, PilA (Fig. 3b), but not FliC, an external protein component of flagella (see Fig. S4c in the supplemental material). We confirmed that SpA binds PilA by coimmunoprecipitation, followed by a Western blot assay for the PilA protein (see Fig. S5b in the supplemental material). The PilA protein was present in our coimmunoprecipitation from two strains producing type IV pili (wild-type PAO1 and PAO1 Δ*pilT*), but absent in negative controls (PAO1 Δ*pilA* and supernatant from PAO1 Δ*pilT* to which SpA was not added). Additionally, binding of SpA to PilA was confirmed by LC-MS/MS analysis of the SpA immunoprecipitate (NSAF of 0.3 in the coimmunoprecipitation sample, and 0.2 in the PAO1 supernatant loading control). In contrast, the LC-MS/MS analysis did not identify FliC as a SpA binding partner (NSAF of 0.03 in the coimmunoprecipitation sample, 0.2 in the PAO1 supernatant loading control), in support of the FITC-SpA experimental results. Finally, we found that *S. aureus* culture supernatant was unable to inhibit binding of FITC-SpA to the cell surface of wild-type MPAO1, which suggests that the number of binding sites for SpA on the *P. aeruginosa* cell surface (including Psl polysaccharide and the PilA protein) was in excess of the amount of SpA present in *S. aureus* supernatant.

### *P. aeruginosa* Psl production impacts biofilm inhibition by *S. aureus* supernatant.

To investigate the genetic relationships between inhibited and uninhibited *P. aeruginosa* isolates, we performed pulsed-field gel electrophoresis (PFGE) on the *P. aeruginosa* isolates (*n* = 16) collected from patient 102 over the course of 2 years. While all of the PFGE patterns among these isolates were highly similar, indicating that they arose from a single lineage, the isolates could be placed into 3 PFGE groups based on a single band shift (see Fig. S6a in the supplemental material). Isolates that did not display biofilm inhibition had one of two patterns (pattern 1, 22 isolates; pattern 2, 2 isolates), and these patterns differed by a downward shift in the largest band. Strains that displayed biofilm inhibition in SA113 Sup exhibited an additional downward shift of this PFGE band compared to pattern 2 isolates (pattern 3, 9 isolates), suggestive of an additional, larger genomic deletion in the *P. aeruginosa* isolates that displayed the biofilm inhibition phenotype.

We hypothesized that inhibited strains were missing genetic material that protects against biofilm inhibition by *S. aureus*. To better define the genetic differences between these isolates and to characterize the genomic deletion suggested by PFGE, we sequenced the genomes of isolates representing each PFGE pattern from patient 102: 102-2 (pattern 1; not inhibited), 102-26 (pattern 2; not inhibited), 102-21 (pattern 3; inhibited), 102-30 (pattern 3; inhibited). In the pattern 3 isolates, we identified a large genomic deletion, encompassing 202 genes corresponding to the subsequent PFGE shift relative to pattern 2 described above (see Fig. S6b in the supplemental material). Based upon our knowledge of genetic determinants that influence *P. aeruginosa* biofilm formation, one set of genes of interest that were absent in the pattern 3 isolates was the entire Psl biosynthetic operon. As expected, these isolates did not produce Psl (see Fig. S6c). Nevertheless, the inhibited isolates still formed pure culture biofilms at levels equivalent to their clonally related, noninhibited, Psl-producing counterparts (see Table S2 in the supplemental material). These data suggest an important role for Psl in the biofilm-inhibitory effect of *S. aureus* supernatant. In support of this hypothesis, the 9 *P. aeruginosa* isolates (collected from 3 different patients) that displayed biofilm inhibition did not produce appreciable amounts of Psl, as measured via Psl immunoblotting (see Table S2).

### Psl protects *P. aeruginosa* from biofilm inhibition by *S. aureus* supernatant.

Nonmucoid *P. aeruginosa* isolates were previously divided into four classes based on their dependencies on EPS for biofilm formation (34). To further investigate the role of Psl production in biofilm inhibition by *S. aureus*, we assayed a subset of *P. aeruginosa* isolates from both clinical and environmental sources that represented each of these four classes. We found that mutants defective for Psl production (Δ*pslD*) were inhibited for biofilm formation regardless of matrix usage class, whereas isogenic wild-type strains that produced Psl were not inhibited (see Table S2 in the supplemental material). Additionally, we found that, whereas a *P. aeruginosa* mutant lacking Psl was inhibited for biofilm formation by *S. aureus* supernatant (MPAO1 Δ*pslD*), a mutation for SpA’s other *P. aeruginosa* cell surface target (type IV pili; MPAO1 Δ*pilA*) was not inhibited in *S. aureus* supernatant (see Fig. S6d in the supplemental material). Finally, we found that preincubating purified SpA with purified Psl abrogated the biofilm-inhibitory effect of SpA on MPAO1 Δ*pslD* in a crystal violet assay (see Fig. S6e). Together with the observations for clinical isolates, these results indicate that Psl protects *P. aeruginosa* from biofilm inhibition by *S. aureus*.

### SpA reduces phagocytosis of *P. aeruginosa* by neutrophils.

**FIG 4:**
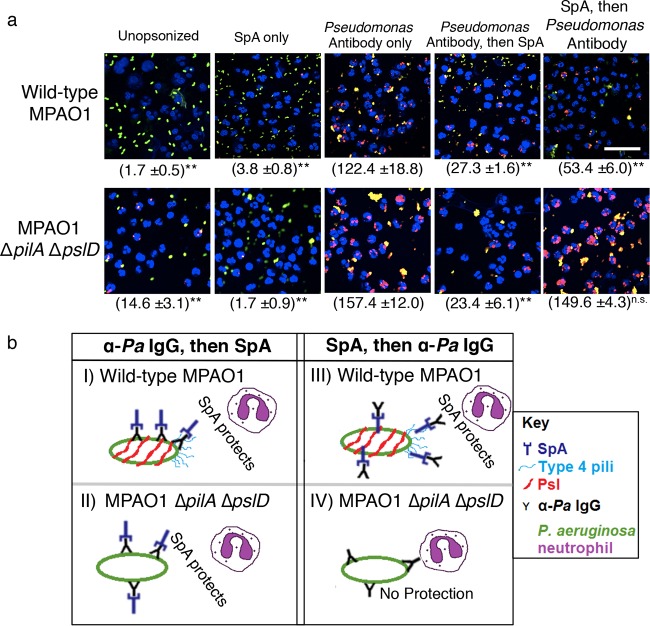
(a) SpA protects *P. aeruginosa* from neutrophil phagocytosis, as shown in these representative images from neutrophil phagocytosis experiments of the indicated *P. aeruginosa* strains, with and without the addition of SpA and/or antipseudomonal antibody. Blue, nuclei; green/yellow, extracellular *P. aeruginosa*; red, internalized *P. aeruginosa*. Asterisks indicate a significant difference in phagocytosis compared to the addition of *Pseudomonas* antibody only (*, *P* < 0.05; **, *P* < 0.001; n.s., no significant difference relative to *Pseudomonas* antibody only). Bar, 20 µm. Magnification (oil objective), ×60. The numbers (means ± standard deviations) of internalized bacteria are indicated in parentheses and are representative of 100 infected cells (neutrophils), examined from triplicate coverslips. (b) Model of two mechanisms of SpA protection of *P. aeruginosa* from IgG-mediated opsonophagocytosis. (I and II) Broad protection for both wild-type MPAO1 and MPAO1 Δ*pilA* Δ*pslD* due to SpA binding to the exposed Fc region of the antipseudomonal (α-*Pa*) IgG. (III) Specific protection of wild-type MPAO1 via binding of SpA to two receptors on the *P. aeruginosa* cell surface (Psl and type 4 pili) prior to opsonization with IgG. (IV) When preincubated with SpA prior to opsonization by IgG, MPAO1 Δ*pilA* Δ*pslD* is not protected from neutrophil phagocytosis, because it is unable to bind SpA on its cell surface.

While the *in vitro* biofilm assay provided a convenient platform for identifying *P. aeruginosa* cell surface targets of SpA binding, and biofilm formation on abiotic surfaces is likely important for some chronic infections, the relevance of this model for CF infections and other chronic infections without abiotic surfaces is less clear. In contrast, each of these chronic infections is characterized by a marked host response. SpA is known to influence host immune responses to *S. aureus*. For example, *S. aureus* surface-associated SpA is known to protect *S. aureus* cells from opsonization by host IgG, and thus from neutrophil phagocytosis (35). SpA binds many mammalian IgG molecules at the nonvariable Fc region (36). Since SpA can bind to at least two abundant *P. aeruginosa* surface structures, Psl and PilA, we hypothesized that extracellular SpA would also protect *P. aeruginosa* from neutrophil phagocytosis by inhibiting opsonization by IgG. To test this hypothesis, we measured neutrophil phagocytosis of *P. aeruginosa* when opsonized with anti-*Pseudomonas* antibody in the presence and absence of SpA (Fig. 4a). We found that the addition of SpA significantly altered IgG-mediated neutrophil phagocytosis; however, further mechanistic insight came from altering the order of addition of antibody and SpA. Opsonization of *P. aeruginosa* with IgG prior to incubating with SpA (followed by washing) led to decreased phagocytosis of wild-type MPAO1 by neutrophils, presumably because exposed Fc receptors on *Pseudomonas*-bound antibody were then “capped” by SpA, preventing uptake. Furthermore, incubation with SpA prior to opsonization (analogous to the effect of membrane-associated SpA on *S. aureus* cells) decreased uptake by neutrophils by approximately 60% in wild-type MPAO1 (the strain that binds the most SpA).

To determine whether SpA protection against neutrophil uptake was mediated by its association with Psl and PilA when preincubated with *P. aeruginosa* prior to antibody challenge, we repeated the phagocytosis experiments with MPAO1 Δ*pslD* Δ*pilA*. Opsonization prior to adding SpA protected this mutant from phagocytosis similar to that for wild-type MPAO1. In contrast with wild-type MPAO1, however, preincubating this mutant with SpA prior to opsonization did not significantly decrease uptake by neutrophils (Fig. 4a and b, image IV), suggesting that this mechanism of protection requires the presence of cell surface binding targets for SpA. Additionally, we performed the same experiment in the MPAO1 Δ*pslD* single-mutant background and again observed no protection from phagocytosis upon preincubation with SpA (data not shown). This observation suggests that Psl is necessary for SpA-mediated protection of *P. aeruginosa* from neutrophil phagocytosis and that the presence of PilA alone on the cell surface is not sufficient for this protection.

Together, these results suggested that SpA inhibited neutrophil phagocytosis of *P. aeruginosa* by at least two mechanisms: (i) by blocking antibody prebound to *P. aeruginosa* from being recognized by neutrophils (Fig. 4b, images I and II) and (ii) analogous to its function on the *S. aureus* cell surface, by binding antibody such that the Fc region was obscured from recognition by neutrophil Fc receptors (Fig. 4b, image III). Therefore, SpA protects at least one bacterial species other than *S. aureus* against IgG-mediated neutrophil phagocytosis when attached to the bacterial cell surface.

## DISCUSSION

We found that the *S. aureus* extracellular adhesin SpA binds to specific cell surface targets of *P. aeruginosa*, impacting its persistence-related behaviors*.* Coinfection with these two organisms is common in CF patients; in a recent study among our local pediatric CF population, 40% of patients were coinfected with *P. aeruginosa* and *S. aureus* over a 2-year period (15). It has been suggested that early infection by *S. aureus* may prime the airway for future infection by *P. aeruginosa* (33, 37). In two separate studies, detection of *S. aureus* was a risk factor for earlier infection with *P. aeruginosa* (38, 39). Our results indicate that *S. aureus* may impact the adhesion and phagocytosis of *P. aeruginosa* in vivo through secreted products, particularly SpA, which is known to be highly expressed during growth of CF *S. aureus* clinical isolates in a medium that mimics CF sputum (40). SpA is an important *S. aureus* virulence factor that is known to play multiple roles in mediating the interaction of *S. aureus* with eukaryotic cell targets within the host environment. This study provides the first evidence of an additional role for extracellular SpA in mediating bacterial interspecies interactions and for influencing the interaction of another bacterial species with the host.

Our results also indicate that SpA interacts with two specific structures on the *P. aeruginosa* cell surface: the Psl polysaccharide and the PilA protein component of type IV pili, both of which are known to be important determinants of *P. aeruginosa* biofilm formation (41, 42). Furthermore, our results demonstrate that SpA inhibits biofilm formation by *P. aeruginosa* strains that do not produce Psl, and our data suggest that SpA may do so by binding to type IV pili in the absence of Psl. Psl and type IV pili are known to affect biofilm formation in complex, connected ways. For example, type IV pili are known to contribute only to the earliest stage of biofilm formation (surface attachment) in strains that do not produce Psl (e.g., PA14) (41), but in Psl-producing strains, type IV pili also contribute to later stages of biofilm formation (43). Therefore, Psl protection against SpA-mediated biofilm inhibition could occur via two different, but not mutually exclusive, mechanisms. First, Psl may mask or otherwise outcompete or prevent SpA binding to PilA, thereby leaving type IV pili free to contribute to biofilm development. Alternatively, the Psl polysaccharide itself may be sufficient to mediate cell attachment to surfaces, independent of SpA binding to type IV pili. *P. aeruginosa* forms different types of biofilms, including surface-associated communities and unattached aggregates. We found that SpA inhibited the former but not the latter type of biofilm. While the relevance of abiotic surface-attached biofilms for CF infections is not known, this observation provided a convenient and sensitive assay for identifying cell surface structures important for the effect of SpA on phagocytosis and, perhaps, other behaviors relevant to chronic infection.

Clinical isolates of *P. aeruginosa* defective for Psl production have not been previously described. However, *P. aeruginosa* populations are known to diversify phenotypically and genotypically during chronic infections, such as in CF infections, often impacting EPS production (44–46). We found that *P. aeruginosa* strains that produce high levels of Psl (rugose small-colony variants [RSCVs] PAO1 Δ*fliC* and PAO1 Δ*wspF*) (see Fig. S5c in the supplemental material) hyperbind extracellular SpA *in vitro*. As clinical isolates with this EPS hyperproduction phenotype are isolated frequently from CF patients (47), it is possible that this interaction with SpA may serve to protect a diverse population of *P. aeruginosa* isolates from opsonophagocytosis in polymicrobial infections. Future work will be required to identify whether *P. aeruginosa* biofilm matrix production phenotypes impact the persistence of one or both species during coinfection and the extent to which an interaction of the Psl polysaccharide with extracellular SpA is involved.

SpA is often described as a multifunctional virulence factor, and among its best-known binding targets is the Fcγ domain of mammalian IgGs. For SpA associated with the *S. aureus* cell surface, Fcγ binding results in coating of the *S. aureus* cell surface with IgG molecules oriented “outward,” such that they cannot bind to the neutrophil Fc receptor (28), preventing Fc receptor-mediated opsonophagocytosis and bacterial killing (48). Here, we found that SpA can perform a similar function for other bacteria: SpA protected *P. aeruginosa* from IgG-mediated neutrophil opsonophagocytosis *in vitro*, and full protection required the presence of both Psl and PilA. SpA’s ability to bind to the Psl polysaccharide suggests that the *P. aeruginosa* EPS matrix could selectively retain SpA. However, the contribution of extracellular SpA to multifactorial behaviors like bacterial persistence, particularly in a polymicrobial community, remains largely understudied.

We found that exogenous addition of SpA subsequent to opsonization of *P. aeruginosa* by IgG impeded phagocytosis by neutrophils regardless of whether the *P. aeruginosa* strain expressed either Psl or type IV pili. Under these conditions, we predict that SpA binds to and blocks the exposed Fc region on IgG, thus rendering it unavailable for binding by a neutrophil Fc receptor, an early step in initiation of IgG-mediated phagocytosis (Fig. 4b, images I and II). It is likely that this protective effect of extracellular SpA is broadly applicable to other bacterial species when coinfecting with *S. aureus* in a polymicrobial infection independent of the bacterium’s ability to bind SpA on its cell surface. Second, and more specific to *P. aeruginosa*, we found that SpA that was bound to the cell surface of *P. aeruginosa* prior to exposure to IgG significantly reduced phagocytosis by neutrophils. We showed that this effect was dependent on the ability of SpA to bind to the cell surface of *P. aeruginosa*: phagocytosis of a strain of *P. aeruginosa* incapable of producing either Psl or type IV pili was unaffected by preincubation with SpA. Therefore, our results suggest that *P. aeruginosa* is able to share the protective benefit of extracellular SpA produced by *S. aureus*, resulting in a reduction in IgG-mediated phagocytosis of *P. aeruginosa* by neutrophils, through at least two mechanisms. While protective *in vitro* interactions between these two species have been previously observed (21, 24, 49), none has involved a manipulation of the host immune response.

In addition to identifying a new interspecies role for SpA, our findings also suggest a novel social role for Psl, in which this EPS interacts with nearby bacterial species through their exoproducts. Specifically, production of Psl determines the ability of a *P. aeruginosa* strain to attach to surfaces and influences neutrophil uptake of opsonized *P. aeruginosa* cells when SpA is present. While SpA bound to the cell surface of *P. aeruginosa* retains at least one important known function, protection from IgG-mediated phagocytosis, further work to define whether SpA retains any of its additional known functions when bound to *P. aeruginosa* (such as binding to von Willebrand factor [50] or tumor necrosis factor-α receptor 1 [51]) will allow us to better understand the full scope of this interspecies interaction and its relevance for a variety of diseases. Given that bacteria rarely exist in isolation, whether in human tissues or in the environment, these results underscore the need for more work to define the collective function of extracellular microbial virulence factors in polymicrobial systems.

## MATERIALS AND METHODS

### Bacterial strains and media.

Bacterial isolates and plasmids used in this study are listed in Table S1 in the supplemental material). Unless otherwise noted, strains were grown at 37°C in Luria-Bertani (LB; Becton, Dickinson) broth buffered with 50 mM morpholinopropanesulfonic acid (MOPS; pH 7.0) (LB-MOPS). Clinical bacterial isolates identified in Table S1 were collected as part of a single-center clinical study of children with CF (15) approved by the Seattle Children’s Hospital IRB (no. 12496).

### Screen for altered biofilm phenotypes in *S. aureus* supernatant.

We screened 24 *P. aeruginosa* clinical isolates from 9 pediatric cystic fibrosis patients for altered biofilm formation in *S. aureus* cell-free culture supernatant, as measured in a crystal violet attachment assay in microtiter plates (Nunc, Thermo Scientific, Waltham, MA). Biofilm “inhibition” was defined as a statistically significant decrease (*P* < 0.05) in biofilm biomass as measured by the CV assay in *S. aureus* SA113 supernatant over the same strain grown in LB-MOPS.

### *S. aureus* supernatant treatment and bioassay-guided fractionation.

*S. aureus* cell-free supernatant was subjected to the following treatments in order to characterize the biofilm inhibition signal: boiling, proteinase K (Sigma-Aldrich, St. Louis, MO), DNase I (NEB, Ipswitch, MA), RNase I (Thermo Scientific, Waltham, MA), and molecular-weight-cutoff filters (Amicon; Sigma-Aldrich, St. Louis, MO). Crystal violet assays were performed with PA102-2 and PA102-21 as previously described, to test for retention of biofilm-inhibitory activity in the supernatant after each treatment. Size-exclusion fast-protein liquid chromatography (FPLC) was performed using a HiPrep 16/60 Sephacryl S200 (GE Healthcare) column, and each fraction was screened for biofilm-inhibitory activity against strain 102-21 in the CV assay. Six inhibitory fractions and three noninhibitory control fractions were chosen for LC-MS/MS.

### Identification of candidate biofilm-inhibitory proteins by mass spectrometry (LC-MS/MS).

Candidate biofilm-inhibitory proteins were identified based on their frequency of detection in active fractions compared to inactive control fractions, as determined based on NSAF, with a focus on predicted extracellular proteins.

### Identification of SpA as a biofilm inhibitor protein.

Cell-free culture supernatant was prepared from SA113 Δ*spa*, and the CV assay was repeated with PA102-2 and PA102-21. Loss of biofilm inhibition activity was defined as a statistically significant increase in PA102-21 biofilm biomass in SA113 Δ*spa* supernatant compared to wild-type SA113 supernatant. Purified SpA (Sigma) was added into LB-MOPS at 10 and 100 µg/ml, and the CV assay was repeated with PA102-2 and PA102-21. Biofilm inhibition activity was defined as a statistically significant decrease in PA102-21 biofilm biomass in wells with purified SpA compared to wells with LB-MOPS alone and compared to wells with 10 or 100 µg/ml bovine serum albumin (negative control).

### Sequencing of 102 series.

Whole-genome sequencing was performed on four clonally related, clinical *P. aeruginosa* isolates: two biofilm-inhibited (102-21 and 102-30) and two noninhibited (102-2 and 102-26) isolates, essentially as previously described (52). Annotated genomes can be viewed at http://tools.nwrce.org/pgat/.

### Psl immunoblot assay.

*P. aeruginosa* isolates were grown in LB-MOPS overnight at 37°C with shaking (225 rpm). The Psl immunoblot assay was performed as previously described (33). To detect Psl in these samples, an anti-Psl antibody cocktail containing 3 monoclonal anti-Psl antibodies (MedImmune, Gaithersburg, MD) was used at a 1:3,000 dilution for 1 h in Tris-buffered saline with Tween 20 (TBST) and 1% milk, followed by a secondary goat anti-human antibody (Abcam, Cambridge, MA) at 1:5,000 for 1 h in TBST.

### FITC-SpA binding assay.

*P. aeruginosa* isolates were grown to mid-log phase in LB-MOPS. FITC-labeled SpA (Sigma-Aldrich, St. Louis, MO) was added to each culture at a final concentration of 100 µg/ml. Cultures were incubated for 10 min at room temperature, then pelleted and washed three times with phosphate-buffered saline (PBS). The cell suspension was transferred to a Costar flat-bottom, black with clear bottom 96-well plate (Sigma-Aldrich, St. Louis, MO). Relative FITC-SpA binding of each strain was determined by calculating the relative fluorescence: the FITC fluorescence (excitation at 495 nm, emission at 519 nm) normalized to the cell density (the optical density at 600 nm [OD_600_]).

### Coimmunoprecipitation results for SpA with PilA and Psl.

Protein G Dynabeads (Life Technologies, Carlsbad, CA) were incubated with 100 µg/ml anti-SpA monoclonal antibody (Sigma-Aldrich, St. Louis, MO) following the manufacturer’s instructions, and then incubated with 100 µg/ml purified protein A (Sigma-Aldrich, St. Louis, MO) for 20 min at 4°C. Beads were washed three times with Tris-buffered saline (TBS) plus 0.1% Tween and resuspended in TBS. To assay for SpA binding to Psl, wild-type MPAO1, MPAO1 Δ*pslD*, or MPAO1 Δ*cdrA* were grown overnight in LB-MOPS. Cultures were normalized for cell density, cells were pelleted, and the supernatant was saved for coimmunoprecipitation with SpA. Triton X-100 was added to each supernatant sample to a final concentration of 0.1% in the supernatant. Coimmunoprecipitation reaction mixtures were incubated at 4°C for 1 h on a low-speed rotator. Beads were washed four times with washing buffer (TBS with 200 mM NaCl and 0.1% Triton X-100), then resuspended in 0.5 M EDTA, pH 8.0. Psl was detected in these samples by performing Psl immunoblot assays as described in the “Psl immunoblot assay” section above.

For the PilA coimmunoprecipitation experiment, the following strains were used: wild-type MPAO1, MPAO1 Δ*pilT*, and MPAO1 Δ*pilA*. Supernatants from overnight cultures of each strain were spiked with 100 µg/ml SpA and incubated on a rotator at 4°C for 1 h. Protein G Dynabeads that had been preloaded with anti-SpA monoclonal antibody were added to each reaction mixture, incubated at 4°C on a rotator for 1 h, then washed and resuspended in Laemmli buffer (Bio-Rad Laboratories, Hercules, CA). Proteins were run on an SDS-PAGE gel (Bio-Rad) and then transferred to a nitrocellulose membrane for Western blotting with anti-PilA polyclonal and an anti-SpA polyclonal antibody (Sigma-Aldrich, St. Louis, MO).

### Identification of SpA binding target on the *P. aeruginosa* cell surface by mass spectrometry (LC-MS/MS).

Coimmunoprecipitations of SpA with *P. aeruginosa* supernatants were performed as described above and samples were run on an SDS-PAGE gel. Bands were cut from the gel, and LC-MS/MS was performed as described above, but using in-gel protein digests. Peptides were identified using the *P. aeruginosa* PAO1 FASTA database. Coimmunoprecipitation pairs with an NSAF score greater than 0.05 were considered SpA binding targets.

### Neutrophil isolation and phagocytosis assays.

Human neutrophils were obtained from healthy adult donors, using an approved IRB protocol (number 2009H0314) at The Ohio State University. Cells were isolated, and phagocytosis assays were performed as previously described (53) using an antipseudomonal antibody and 100 µg/ml SpA.

Further details on our methods are described in Text S1 in the supplemental material.

## SUPPLEMENTAL MATERIAL

Text S1  Supplemental methods and references. Download Text S1, DOCX file, 0.1 MB

Figure S1 Analysis of SA113 supernatant by chemical and physical treatments indicate that biofilm inhibition activity is due to a protein larger than 30 kDa. In each case, treated supernatants were added to a Psl^−^ CF clinical isolate (102-21) and biofilm formation assayed as described in Text S1. Asterisks indicate statistically significant increases in biofilm biomass compared to growth in untreated SA113 supernatant (*P* < 0.05) and loss of biofilm inhibition. Download Figure S1, TIF file, 0.7 MB

Figure S2  Screen for FPLC fraction activity in a Psl^−^ clinical isolate (102-21), based on crystal violet (CV) staining (4 h). *, active biofilm inhibitor fractions chosen for LC-MS/MS; **, inactive fractions, to compare relative abundance of candidate inhibitor proteins. Download Figure S2, TIF file, 1.3 MB

Figure S3 Crystal violet assay (4-h) screen of supernatant from NARSA collection transposon mutants of all available MSCRAMMs in the JE2 background, performed on a Psl^−^ *P. aeruginosa* clinical isolate (102-21). A transposon insertion in the *spa* gene resulted in a loss of the biofilm inhibition phenotype. An asterisk indicates a significant increase in biofilm biomass above that of the LB control or wild-type JE2 supernatant (*P* < 0.05), suggesting a loss of the biofilm inhibition phenotype due to transposon insertion. Download Figure S3, TIF file, 0.9 MB

Figure S4  (a) The Psl-producing clinical isolate 102-2 binds more FITC-SpA than the clonally related Psl nonproducer, 102-21. *, *P* < 0.001 compared to wild-type PAO1 or MPAO1. (b) PAO1 Δ*fliC* is an RSCV that is commonly isolated from CF patient sputum and hyperbinds FITC-SpA in a Psl-dependent manner. (c) PAO1 Δ*wspF*, another common CF RSCV that is known to overproduce Psl, also hyperbinds SpA in a Psl-dependent manner. Download Figure S4, TIF file, 2.4 MB

Figure S5  (a) Coimmunoprecipitation experiments with *P. aeruginosa* culture supernatants followed by Psl immunoblotting. SpA bound to beads was incubated with culture supernatants from the indicated *P. aeruginosa* strains, followed by centrifugation, washing, and dot blotting with anti-PsI (α-Psl) antibody. Supernatant samples in the bottom row differed from those in the top row in that they had been boiled for 25 min to denature any proteins prior to coimmunoprecipitation. Data for the SpA beads-only control indicate results with beads with both anti-SpA (α-SpA) antibody and bound SpA, to control for cross-reactivity of the anti-Psl antibody with SpA-coated beads. The SpA antibody (Ab) control entailed beads with anti-SpA antibody, incubated in wild-type MPAO1 culture supernatant in the absence of SpA to control for nonspecific binding of Psl by the anti-SpA antibody. Densitometry values (in parentheses) were measured with the ImageJ software. Load, the supernatant sample used for coimmunoprecipitation. (b) Coimmunoprecipitation experiments of the indicated *P. aeruginosa* culture supernatants, performed as for panel a, except with Western blotting using anti-PilA antibody. The beads-only control represents beads bound to anti-SpA antibody but without SpA, incubated in wild-type PAO1 supernatant to control for nonspecific binding of PilA to beads. PilT deletion overproduces PilA. The PilA deletion strain was a negative control to rule out that the PilA antibody produces a false-positive signal. Densitometry (values in parentheses) was performed to quantify the amount of protein in the PilA coimmunoprecipitation lane, normalized to the amount of SpA. Load, a loading control, i.e., the supernatant sample input into the coimmunoprecipitation. Download Figure S5, TIF file, 1.1 MB

Figure S6  (a) *P. aeruginosa* isolates collected from patient 102 over 8.3 months displayed a total of three different PFGE patterns. Earlier isolates exhibited pattern 1; subsequent isolates displayed patterns 2 and 3, which differed according to the presence or absence of larger DNA fragments. (b) Map of chromosomal deletions in 102-2 (PFGE pattern 1), 102-26 (pattern 2), and 102-21/102-30 (pattern 3), relative to the PAO1 genome, from whole-genome sequencing data. The entire Psl operon is deleted in pattern 3 isolates. (c) Psl dot blot assay results, showing that isolates with patterns 1 and 2 produce Psl (102-2 and 102-26), whereas two isolates with pattern 3 do not (102-21 and 102-30). (d) Crystal violet assay results, demonstrating that an MPAO1 Δ*pilA* mutant was not inhibited by *S. aureus* cell-free culture supernatant, whereas a Psl mutant in the same genetic background (MPAO1 Δ*pslD*) was inhibited. Crystal violet-stained biofilms indicate results after 4 h of growth. For each pair of bars in the graph, the left bar shows growth in L and the right bar shows growth in SA113 supernatant. (e) Psl protects *P. aeruginosa* MPAO1 Δ*pslD* from SpA-mediated biofilm inhibition. Crystal violet assay results demonstrated that preincubation of 10 µg/ml purified SpA with 100 µg/ml purified Psl abrogated the biofilm-inhibitory effect of SpA on *P. aeruginosa* Δ*pslD*. SpA-only and Psl-only controls included concentrations of 10 µg/ml and 100 µg/ml, respectively. *, *P* < 0.05 compared to biofilm biomass in the LB control; n.s., no statistically significant difference in biofilm biomass relative to the LB control. Download Figure S6, TIF file, 2.6 MB

Table S1 Strains, plasmids, and primers used in this study. Table S1, DOCX file, 0.1 MB

Table S2 Results of the 4-h biofilm formation assay, measured via crystal violet staining of patient 102 isolates, isolates from 8 additional CF patients, and wild-type and isogenic *pslD* mutants from the four biofilm matrix usage classes defined by Colvin et al. (2011). (Roman numerals in parentheses next to strain names indicate the matrix usage class for defined strains.) Table S2, DOCX file, 0.1 MB

Table S3  Results of the 4-h biofilm formation assay, measured via crystal violet staining of a representative *P. aeruginosa* clinical isolate (102-21), showing that the biofilm inhibition phenotype occurs in supernatant from multiple common lab strains and clinical isolates of *S. aureus*. (Strains 102-10 and 102-12 are *S. aureus* clinical isolates that were collected from patient 102, from whom the *P. aeruginosa* isolate 102-21 was also collected.) Table S3, DOCX file, 0.1 MB
