# Supplementary material for: Staphylococcus aureus Protein A Mediates Interspecies Interactions at the Cell Surface of Pseudomonas aeruginosa
Source: mBio. 2016 May 24;7(3):e00538-16. doi: 10.1128/mBio.00538-16 (PMC4895107; doi:10.1128/mBio.00538-16)
Supplement: Text S1 — Supplemental methods and references. Download [file mbo003162819s1.docx]

**Supplemental Materials and Methods**

Bacterial Strains and Media

For plasmid selection, 10µg/ml erythromycin (Sigma-Aldrich, St. Louis, MO) or 10 or 12.5µg/ml chloramphenicol (Sigma-Aldrich, St. Louis, MO) was used with *S. aureus* and12.5µg/ml chloramphenicol was used with *Escherichia coli*.

Screen for Altered Biofilm Phenotypes in *S. aureus* Supernatant

*S. aureus* SA113 was inoculated to an OD_600_ of 0.05 from mid-log cells and grown shaking at 37^o^C for 6 hours in LB broth buffered with 50mM MOPS (LB MOPS). Supernatant was harvested by pelleting cells in a centrifuge at 4^o^C and filter-sterilizing the supernatant with a 0.22 µm low protein binding filter. CV attachment assays were performed in 96 well plates using mid-log *P. aeruginosa* diluted to an OD_600_ of 0.05 in either LB MOPS or in SA113 cell-free supernatant. Plates were incubated at 37^o^C for 2, 4, or 6 hours without shaking.

To assay for biofilm formation, the liquid culture was removed without disturbing the pellicle and wells were rinsed gently with ddH_2_O to remove planktonic cells. After staining for 10 minutes with 0.1% crystal violet, wells were rinsed gently with ddH_2_O. Crystal violet was eluted from the biofilm biomass with 95% ethanol for 10 minutes, then transferred into a new 96 well plate and absorbance read at 595nm. Six replicate wells were used for each condition. For supplemental figure S.6e, purified SpA and purified Psl were pre-incubated in sterile water for 30 minutes at room temperature on a rotator, then added at a final concentration of 10μg/mL SpA and 100μg/mL Psl into LB MOPS for the crystal violet assay.

DNA Macrorestriction Analysis of Patient 102 *P. aeruginosa* Isolates

Genetic relatedness of the clinical isolates was determined by Pulsed-field Gel Electrophoresis (PFGE) according to the Centers for Disease Control and Prevention PulseNet protocol(59). Chromosomal DNA was digested with 30 U of *Spe*I (Roche) at 37°C for 16 hr. The DNA restriction fragments were separated in a 1% SeaKem Gold agarose gel using a CHEF DR-III (Bio-Rad) with the following conditions: field strength 6 V/cm, angle 120°, initial switch time 1 s, final switch time 35 s, total run time 23 hr. at 14°C. DNA restriction patterns were analyzed using BioNumerics software (v. 6.5; Applied Maths, Sint-Martens-Latem, Belgium). The dendrogram was constructed by the UPGMA (Unweighted Pair Group Method with Arithmetic mean) clustering method and Dice similarity coefficients with an optimization setting of 0.5% and position tolerance of 1.0%.

Whole Genome Sequencing of Patient 102 *P. aeruginosa* isolates

For each genome either a random-fragment library was constructed using a custom paired-end protocol(60) or standard Illumina Nextera libraries were constructed according to manufacturer’s guidelines (Illumina Inc., San Diego, CA). Paired-end libraries for each genome were used to generate 76 bp or 100 bp reads with the Illumina GAIIx or HiSeq 2000, respectively (coverage > 100 reads/genomic position). Sequencing of libraries was performed according to manufacturer’s standards (Illumina Inc., San Diego, CA). Draft genome assemblies were generated with ABySS 1.3.4(61) using the complete PAO1 genome (NC_002516) as a reference. Genomes were annotated using the Prokaryotic Genome Analysis Tool (PGAT)(62), which grouped all genes into orthologous gene families (genes within a family share at least 96% homology and 80% coverage of the total gene sequence). Gene family annotation was based on previously annotated genomes, including PAO1, PA14 and LESB58, when available. Open reading frames (ORFs) in regions lacking known genes were predicted using Prodigal(63), and new gene families were annotated based on searches in the National Center for Biotechnology Information (NCBI) Conserved Domain Database (CDD)(64). Annotated genomes can be viewed at <http://tools.nwrce.org/pgat/>.

Bioassay-guided Fractionation of *S. aureus* Supernatant

*S. aureus* cell-free supernatant was subjected to the following treatments in order to characterize the biofilm inhibition signal: boiled for 10 minutes in a water bath at 100^o^C, 0.5mg/mL Proteinase K at 37^o^C for 2 hours, 1 U/mL Dnase I at 37^o^C for 2 hours, 4μg/mL Rnase I at 37^o^C for 2 hours, flow through 3kDa molecular weight cutoff filter, retained in 30kDa molecular weight cutoff filter. Each molecular weight cutoff filter fraction was brought up to the starting volume with clean LB MOPS. CV assays were performed with PA102-2 and PA102-21 as previously described.

Size exclusion fast protein liquid chromatography (FPLC) was performed using a HiPrep 16/60 Sephacryl S200 (GE Healthcare) column, equilibrated with LB MOPS, pH 7.0. *S. aureus* cell-free supernatant was fractionated with a 3kDa MWCO filter and the fraction containing molecules >3kDa was resuspended in LB MOPS to one half of the initial volume. Five milliliters of this concentrated sample was applied onto the gel filtration column and eluted in LB MOPS at a flow-rate of 0.5 ml/min at 4^o^C, with 4mL per fraction. Each fraction was screened for biofilm-inhibitory activity against PA102-21 in the CV assay. Inhibitory fractions were defined as having an average biofilm biomass (OD_595_) of less than 1 standard deviation below the mean biofilm biomass of all supernatant fractions. Six inhibitory fractions and three non-inhibitory control fractions were chosen for LC-MS/MS.

Identification of Candidate Biofilm-Inhibitory Proteins by Mass Spectrometry (LC-MS/MS)

Total protein in each of the 9 fractions from size exclusion FPLC were denatured in RapiGest (Waters), reduced, alkylated, and digested with trypsin. One microgram of each sample was loaded onto a 75µm inner diameter fused silica capillary (Polymicro) loaded with 30cm of Jupiter Proteo 5 µm reverse-phase material (Phenomenex). Nanoflow liquid chromatography was performed using the Waters nanoACQUITY UPLC system, eluting the peptides over a 180 minute gradient of increasing acetonitrile at a flow rate of 250nL/min. Peptides were ionized via electrospray ionization with a home-built ESI source and analyzed with a Thermo LTQ-FT mass spectrometer. Accurate mass and charge predictions were determined for MS/MS fragmentation spectra using Hardklör and Bullseye(65) algorithms. SEQUEST 2.7 software(66) was used to identify peptides against the *S. aureus* NCTC 8325 FASTA database (SA113 parental strain) and false discovery rates were determined via a decoy database using Percolator(67). Peptides were assembled into protein identifications using ID Picker(68).

Construction of SA113 Δ*spa*

Chromosomal DNA upstream and downstream of *S. aureus* SA113 or HG003 *spa* was amplified via PCR using primers spaUpF01, spaUpR01, spaDownF01, and spaDownR01, then used as a template for SOE-PCR using primers spaUpF01 and spaDownR01. The *spa* deletion construct was cloned into the KpnI and NotI sites of the temperature sensitive vector pIMAY, creating plasmid pIMAY::Δ*spa* (spa deletion construct), then transformed into *E. coli* DH10B cells. The *spa* deletion construct was purified from DH10B cells, transformed into *S. aureus* RN4220, and maintained in RN4220 grown on BHI with 10ug/mL chloramphenicol at 30^o^C. Cell lysates were prepared for transduction of the *spa* deletion construct into strain SA113 using bacteriophage phi-11(69). Following passage at the permissive temperature of 30°C, colonies were screened for chromosomal integration of pIMAY::Δ*spa* by growing at 37°C with 10ug/mL chloramphenicol and PCR with MCS primers IM151 and IM152 to verify that colonies did not contain replicating pIMAY plasmid. To select for double recombination and excision of the pIMAY plasmid backbone from the chromosome, cells were grown overnight in BHI at 30^o^C, then screened for growth on BHI + 1ug/mL Anhydrotetracycline (Atc), but sensitivity to killing on BHI + 10ug/mL chloramphenicol. Deletion of *spa* was confirmed by PCR and sequencing with primers spaUPF-SEQ and spaDOWNR-SEQ.

Co-immunoprecipitation of SpA with Psl polysaccharide

For experiments with boiled *P. aeruginosa* supernatants, supernatant was taken from overnight cultures of wild type MPAO1, MPAO1 Δ*pslD*, and MPAO1 Δ*cdrA* grown in LB with 50mM MOPS. Supernatant samples were aliquoted into duplicate samples, then one set of samples was boiled for 25 minutes at 100^o^C. Co-immunoprecipitations with purified SpA and Psl immunoblots were then performed as described under “Co-immunoprecipitations of SpA with PilA and Psl” in the main text.

Neutrophil Isolation and Phagocytosis Assay

Bacteria were taken from overnight plates, washed with PBS, and opsonized with anti-Pseudomonal antibody that was generated against whole surface antigens of PAO1 (1:100), either before or after 10 minute incubation with 100μg/mL purified SpA. Bacteria–neutrophil association was examined by confocal microscopy (Olympus FV 1000) using a 60× oil objective to directly visualize interactions between *P. aeruginosa* and neutrophils as previously described(58). Attached *P. aeruginosa* was distinguished from internalized bacteria, as *P. aeruginosa* cells attached to neutrophils and stained by fluorescently-labeled anti-Pseudomonal antibodies before permeabilization of neutrophils stained green, whereas internalized bacteria are counterstained red after neutrophil permeabilization. Image analysis software was used to count cell-associated versus internalized *P. aeruginosa* bacteria on 100 randomly selected neutrophils, and compared within each strain of *P. aeruginosa* (with versus without SpA pre-treatment), in three biological replicates.

**Supplemental References**

1. **Ribot EM**, **Fair MA**, **Gautom R**, **Cameron DN**, **Hunter SB**, **Swaminathan B**, **Barrett TJ**. 2006. Standardization of pulsed-field gel electrophoresis protocols for the subtyping of Escherichia coli O157:H7, Salmonella, and Shigella for PulseNet. Foodborne Pathog Dis **3**.

2. **Hayden HS**, **Lim R**, **Brittnacher MJ**, **Sims EH**, **Ramage ER**, **Fong C**, **Wu Z**, **Crist E**, **Chang J**, **Zhou Y**, **Radey M**, **Rohmer L**, **Haugen E**, **Gillett W**, **Wuthiekanun V**, **Peacock SJ**, **Kaul R**, **Miller SI**, **Manoil C**, **Jacobs MA**. 2012. Evolution of Burkholderia pseudomallei in Recurrent Melioidosis. PLoS ONE **7**:e36507.

3. **Simpson JT**, **Wong K**, **Jackman SD**, **Schein JE**, **Jones SJM**, **Birol İ**. 2009. ABySS: A parallel assembler for short read sequence data. Genome Res **19**:1117–1123.

4. **Brittnacher MJ**, **Fong C**, **Hayden HS**, **Jacobs MA**, **Radey M**, **Rohmer L**. 2011. PGAT: a multistrain analysis resource for microbial genomes. Bioinformatics **27**:2429–2430.

5. **Hyatt D**, **Chen G-L**, **LoCascio P**, **Land M**, **Larimer F**, **Hauser L**. 2010. Prodigal: prokaryotic gene recognition and translation initiation site identification. BMC Bioinformatics **11**:119.

6. **Marchler-Bauer A**, **Lu S**, **Anderson JB**, **Chitsaz F**, **Derbyshire MK**, **DeWeese-Scott C**, **Fong JH**, **Geer LY**, **Geer RC**, **Gonzales NR**, **Gwadz M**, **Hurwitz DI**, **Jackson JD**, **Ke Z**, **Lanczycki CJ**, **Lu F**, **Marchler GH**, **Mullokandov M**, **Omelchenko MV**, **Robertson CL**, **Song JS**, **Thanki N**, **Yamashita RA**, **Zhang D**, **Zhang N**, **Zheng C**, **Bryant SH**. 2010. CDD: a Conserved Domain Database for the functional annotation of proteins. Nucleic Acids Res **39**:D225–D229.

7. **Hsieh EJ**, **Hoopmann MR**, **MacLean B**, **MacCoss MJ**. 2009. Comparison of Database Search Strategies for High Precursor Mass Accuracy MS/MS Data. J Proteome Res **9**:1138–1143.

8. **Eng JK**, **McCormack AL**, **Yates III JR**. 1994. An approach to correlate tandem mass spectral data of peptides with amino acid sequences in a protein database. J Am Soc Mass Spectrom **5**:976–989.

9. **Kall L**, **Canterbury JD**, **Weston J**, **Noble WS**, **MacCoss MJ**. 2007. Semi-supervised learning for peptide identification from shotgun proteomics datasets. Nat Meth **4**:923–925.

10. **Zhang B**, **Chambers MC**, **Tabb DL**. 2007. Proteomic Parsimony through Bipartite Graph Analysis Improves Accuracy and Transparency. J Proteome Res **6**:3549–3557.

11. **Novick RP**. 1991. [27] Genetic systems in Staphylococci, p. 587–636. *In* Jeffrey H. Miller (ed.), Methods in Enzymology. Academic Press.

12. **Mishra M**, **Byrd MS**, **Sergeant S**, **Azad AK**, **Parsek MR**, **McPhail L**, **Schlesinger LS**, **Wozniak DJ**. 2012. Pseudomonas aeruginosa Psl polysaccharide reduces neutrophil phagocytosis and the oxidative response by limiting complement-mediated opsonization. Cell Microbiol **14**:95–106.
