# Supplementary figures and images for: Staphylococcus aureus Protein A Mediates Interspecies Interactions at the Cell Surface of Pseudomonas aeruginosa
Source: mBio. 2016 May 24;7(3):e00538-16. doi: 10.1128/mBio.00538-16 (PMC4895107; doi:10.1128/mBio.00538-16)

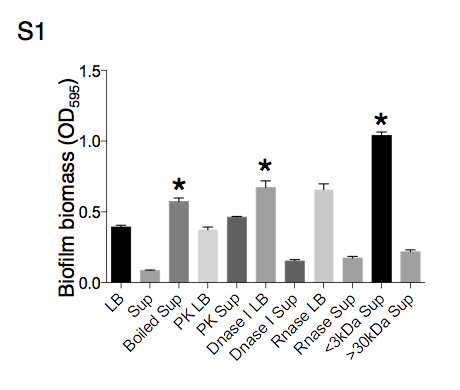

Supplement: Figure S1 — Analysis of SA113 supernatant by chemical and physical treatments indicate that biofilm inhibition activity is due to a protein larger than 30 kDa. In each case, treated supernatants were added to a Psl− CF clinical isolate (102-21) and biofilm formation assayed as described in Text S1. Asterisks indicate statistically significant increases in biofilm biomass compared to growth in untreated SA113 supernatant (P < 0.05) and loss of biofilm inhibition. Download [file mbo003162819sf1.tif]

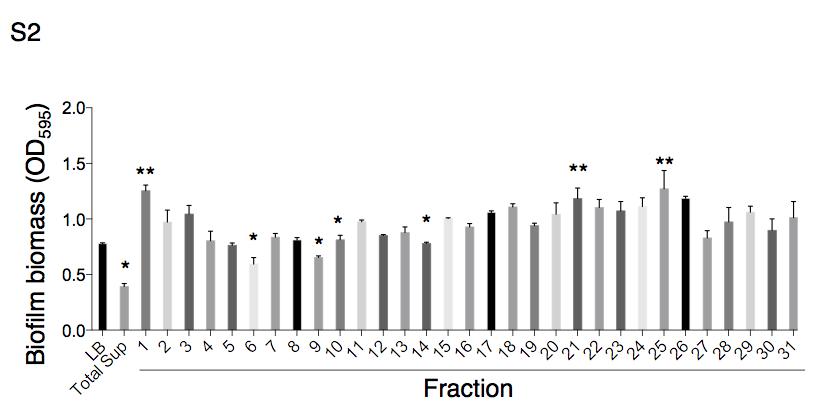

Supplement: Figure S2 — Screen for FPLC fraction activity in a Psl− clinical isolate (102-21), based on crystal violet (CV) staining (4 h). *, active biofilm inhibitor fractions chosen for LC-MS/MS; **, inactive fractions, to compare relative abundance of candidate inhibitor proteins. Download [file mbo003162819sf2.tif]

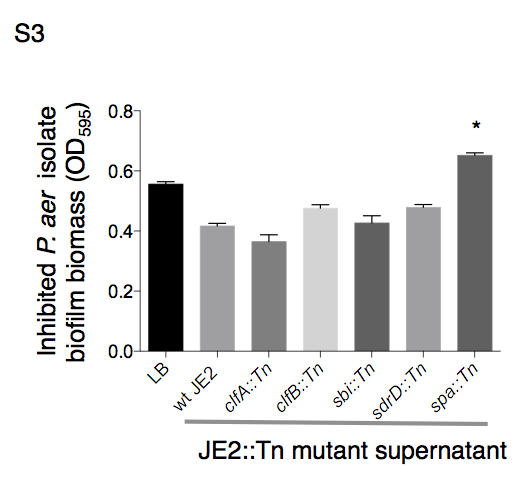

Supplement: Figure S3 — Crystal violet assay (4-h) screen of supernatant from NARSA collection transposon mutants of all available MSCRAMMs in the JE2 background, performed on a Psl− P. aeruginosa clinical isolate (102-21). A transposon insertion in the spa gene resulted in a loss of the biofilm inhibition phenotype. An asterisk indicates a significant increase in biofilm biomass above that of the LB control or wild-type JE2 supernatant (P < 0.05), suggesting a loss of the biofilm inhibition phenotype due to transposon insertion. Download [file mbo003162819sf3.tif]

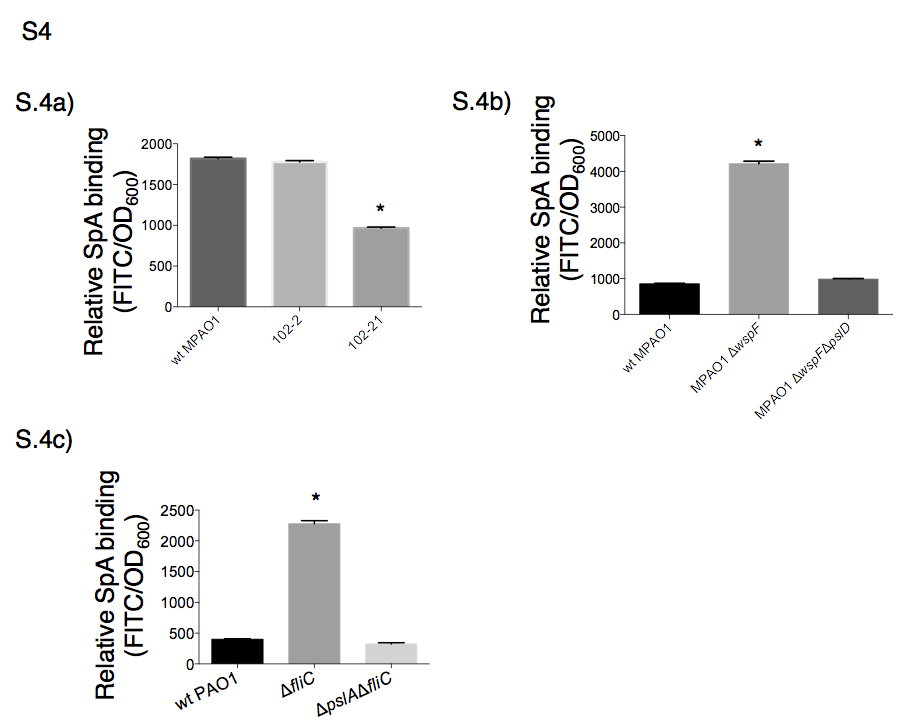

Supplement: Figure S4 — (a) The Psl-producing clinical isolate 102-2 binds more FITC-SpA than the clonally related Psl nonproducer, 102-21. *, P < 0.001 compared to wild-type PAO1 or MPAO1. (b) PAO1 ΔfliC is an RSCV that is commonly isolated from CF patient sputum and hyperbinds FITC-SpA in a Psl-dependent manner. (c) PAO1 ΔwspF, another common CF RSCV that is known to overproduce Psl, also hyperbinds SpA in a Psl-dependent manner. Download [file mbo003162819sf4.tif]

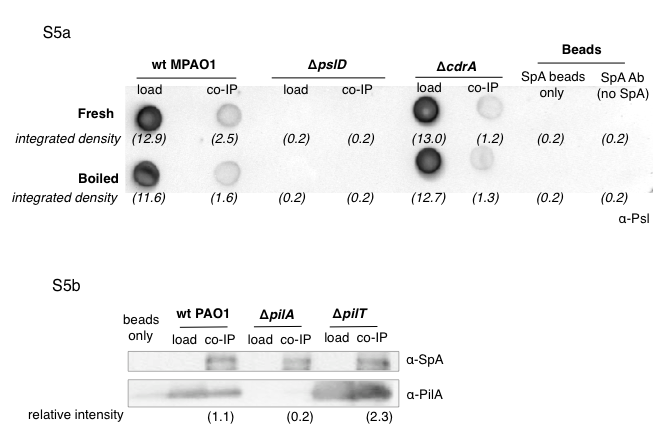

Supplement: Figure S5 — (a) Coimmunoprecipitation experiments with P. aeruginosa culture supernatants followed by Psl immunoblotting. SpA bound to beads was incubated with culture supernatants from the indicated P. aeruginosa strains, followed by centrifugation, washing, and dot blotting with anti-PsI (α-Psl) antibody. Supernatant samples in the bottom row differed from those in the top row in that they had been boiled for 25 min to denature any proteins prior to coimmunoprecipitation. Data for the SpA beads-only control indicate results with beads with both anti-SpA (α-SpA) antibody and bound SpA, to control for cross-reactivity of the anti-Psl antibody with SpA-coated beads. The SpA antibody (Ab) control entailed beads with anti-SpA antibody, incubated in wild-type MPAO1 culture supernatant in the absence of SpA to control for nonspecific binding of Psl by the anti-SpA antibody. Densitometry values (in parentheses) were measured with the ImageJ software. Load, the supernatant sample used for coimmunoprecipitation. (b) Coimmunoprecipitation experiments of the indicated P. aeruginosa culture supernatants, performed as for panel a, except with Western blotting using anti-PilA antibody. The beads-only control represents beads bound to anti-SpA antibody but without SpA, incubated in wild-type PAO1 supernatant to control for nonspecific binding of PilA to beads. PilT deletion overproduces PilA. The PilA deletion strain was a negative control to rule out that the PilA antibody produces a false-positive signal. Densitometry (values in parentheses) was performed to quantify the amount of protein in the PilA coimmunoprecipitation lane, normalized to the amount of SpA. Load, a loading control, i.e., the supernatant sample input into the coimmunoprecipitation. Download [file mbo003162819sf5.tif]

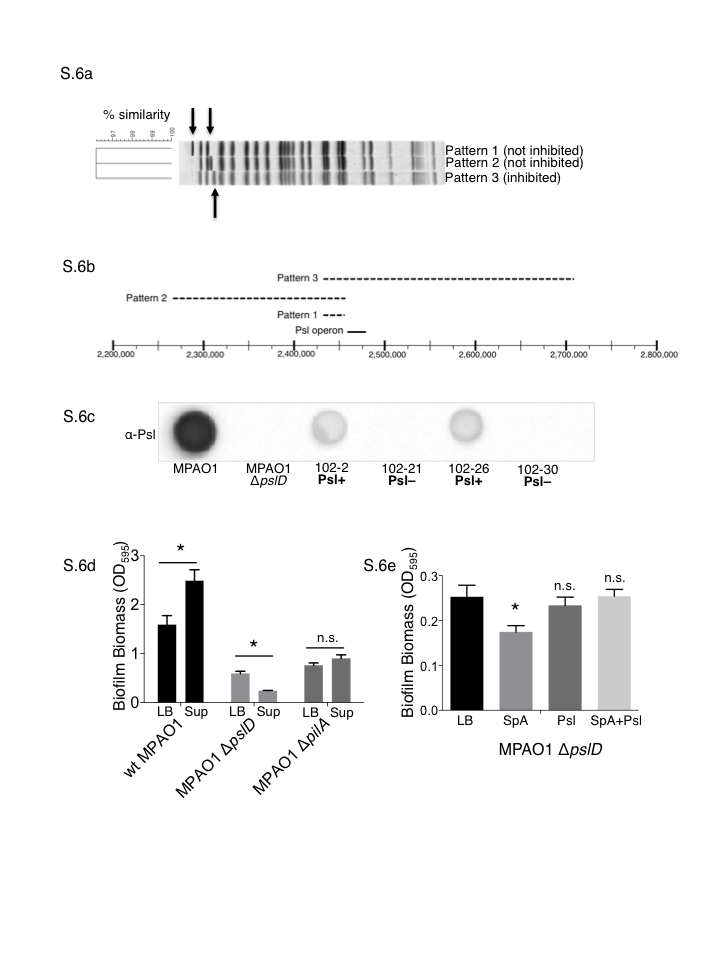

Supplement: Figure S6 — (a) P. aeruginosa isolates collected from patient 102 over 8.3 months displayed a total of three different PFGE patterns. Earlier isolates exhibited pattern 1; subsequent isolates displayed patterns 2 and 3, which differed according to the presence or absence of larger DNA fragments. (b) Map of chromosomal deletions in 102-2 (PFGE pattern 1), 102-26 (pattern 2), and 102-21/102-30 (pattern 3), relative to the PAO1 genome, from whole-genome sequencing data. The entire Psl operon is deleted in pattern 3 isolates. (c) Psl dot blot assay results, showing that isolates with patterns 1 and 2 produce Psl (102-2 and 102-26), whereas two isolates with pattern 3 do not (102-21 and 102-30). (d) Crystal violet assay results, demonstrating that an MPAO1 ΔpilA mutant was not inhibited by S. aureus cell-free culture supernatant, whereas a Psl mutant in the same genetic background (MPAO1 ΔpslD) was inhibited. Crystal violet-stained biofilms indicate results after 4 h of growth. For each pair of bars in the graph, the left bar shows growth in L and the right bar shows growth in SA113 supernatant. (e) Psl protects P. aeruginosa MPAO1 ΔpslD from SpA-mediated biofilm inhibition. Crystal violet assay results demonstrated that preincubation of 10 µg/ml purified SpA with 100 µg/ml purified Psl abrogated the biofilm-inhibitory effect of SpA on P. aeruginosa ΔpslD. SpA-only and Psl-only controls included concentrations of 10 µg/ml and 100 µg/ml, respectively. *, P < 0.05 compared to biofilm biomass in the LB control; n.s., no statistically significant difference in biofilm biomass relative to the LB control. Download [file mbo003162819sf6.tif]
