# Supplementary material for: Staphylococcus aureus Protein A Mediates Interspecies Interactions at the Cell Surface of Pseudomonas aeruginosa
Source: mBio. 2016 May 24;7(3):e00538-16. doi: 10.1128/mBio.00538-16 (PMC4895107; doi:10.1128/mBio.00538-16)
Supplement: Table S1 — Strains, plasmids, and primers used in this study. [file mbo003162819st1.docx]

Supplemental Table 1. Strains, plasmids and primers used in this study.

| ***P. aeruginosa* Strains** | | **Reference** |
| --- | --- | --- |
| MPAO1 | wild-type | Jacobs, 2003 |
| MPAO1Δ*pslD* | *pslD* nonpolar mutant of MPAO1 | Provided by J Harrison^a^ |
| MPAO1Δ*pelC*Δ*pslD* | *pelC pslD* nonpolar mutant of MPAO1 | Provided by J Harrison^a^ |
| MPAO1Δ*pilA*Δ*pslD* | *pilA pslD* nonpolar mutant of MPAO1 | Provided by J Harrison^a^ |
| MPAO1 Δ*pilT* | *pilT* nonpolar mutant of MPAO1 | Provided by J Harrison^a^ |
| PAO1 P_BAD_-pel | chromosomal replacement of the native promoter with *araC*-P_BAD_ promoter | Provided by J Harrison^a^ |
| PAO1 P_BAD_-psl | chromosomal replacement of the native promoter with *araC*-P_BAD_ promoter | Provided by J Harrison^a^ |
| PA14 | wild type | Rahme, 1995 |
| E2 | wild type | Wolfgang, 2003 |
| E2 Δ*pslD* | *pslD* nonpolar mutant of E2 | Colvin, 2012 |
| MSH3 | wild type | Wolfgang, 2003 |
| MSH3 Δ*pslD* | *pslD* nonpolar mutant of MSH3 | Colvin, 2012 |
| CF127 | wild type | Wolfgang, 2003 |
| CF127 Δ*pslD* | *pslD* nonpolar mutant of CF127 | Colvin, 2012 |
| 102-2 | CF patient 102 sputum isolate | This Study^b^ |
| 102-5a | CF patient 102 sputum isolate | This Study^b^ |
| 102-5b | CF patient 102 sputum isolate | This Study^b^ |
| 102-6 | CF patient 102 sputum isolate | This Study^b^ |
| 102-7 | CF patient 102 sputum isolate | This Study^b^ |
| 102-8 | CF patient 102 sputum isolate | This Study^b^ |
| 102-20 | CF patient 102 sputum isolate | This Study^b^ |
| 102-21 | CF patient 102 sputum isolate | This Study^b^ |
| 102-25 | CF patient 102 sputum isolate | This Study^b^ |
| 102-26 | CF patient 102 sputum isolate | This Study^b^ |
| 102-30 | CF patient 102 sputum isolate | This Study^b^ |
| 102-34 | CF patient 102 sputum isolate | This Study^b^ |
| 102-35 | CF patient 102 sputum isolate | This Study^b^ |
| 102-36 | CF patient 102 sputum isolate | This Study^b^ |
| 102-39 | CF patient 102 sputum isolate | This Study^b^ |
| 102-40 | CF patient 102 sputum isolate | This Study^b^ |
| 115-7 | CF patient 115 sputum isolate | This Study^b^ |
| 151-10 | CF patient 151 sputum isolate | This Study^b^ |
| 159-1 | CF patient 159 sputum isolate | This Study^b^ |
| 71-22 | CF patient 71 sputum isolate | This Study^b^ |
| 200-4 | CF patient 200 sputum isolate | This Study^b^ |
| 134-2 | CF patient 134 sputum isolate | This Study^b^ |
| 141-2 | CF patient 141 sputum isolate | This Study^b^ |
| 166-1 | CF patient 166 sputum isolate | This Study^b^ |
|  |  |  |
| ***S. aureus* Strains** |  |  |
| SA113 | wild type | Iordanescu, 1976 |
| SA113 Δ*spa* | *spa* nonpolar mutant of SA113 | This Study |
| HG003 | wild type | Herbert, 2010 |
| HG003 Δ*spa* | *spa* nonpolar mutant of HG003 | This Study |
| *S. aureus* ATCC29213 | wild type | Kim, 1997 |
| *S. aureus* Newman | wild type | Duthie, 1952 |
| SA47-34 | CF patient 47 sputum isolate | This Study^b^ |
| SA102-10 | CF patient 102 sputum isolate | This Study^b^ |
| SA37-12 | CF patient 37 sputum isolate | This Study^b^ |
| SA134-5 | CF patient 134 sputum isolate | This Study^b^ |
| SA102-12 | CF patient 102 sputum isolate | This Study^b^ |
| SA23-71 | CF patient 123 sputum isolate | This Study^b^ |
| SA200-3 | CF patient 200 sputum isolate | This Study^b^ |
| JE2 | Wild type | Fey, 2013 |
| JE2 Tn::*sbi* | Transposon inserted into *sbi* | Fey, 2013 |
| JE2 Tn::*clfA* | Transposon inserted into *clfA* | Fey, 2013 |
| JE2 Tn::*clfB* | Transposon inserted into *clfB* | Fey, 2013 |
| JE2 Tn::*sdrD* | Transposon inserted into *sdrA* | Fey, 2013 |
| JE2 Tn::*spa* | Transposon inserted into *spa* | Fey, 2013 |
| RN4220 pIMAY:: Δ*spa* | restriction defective strain carrying plasmid with *spa* deletion allele | This Study |
|  |  |  |
| ***E. coli* Strains** |  |  |
| DH10B pIMAY:: Δ*spa* | cloning strain carrying plasmid with *spa* deletion allele | This Study |
|  |  |  |
| **Primers** |  |  |
| spaUpF01 | ATATGGTACCGTTTTGTAGAATTCACA ATTCTAGC |  |
| spaUpR01 | GTATTGTTTGTTTTTACAAATTAATACCCCCTGTATGTAT |  |
| spaDownF01 | GGGGTATTAATTTGTAAAAACAAACAATACACAACGATAG |  |
| spaDownR01 | ATATGCGGCCGCGAAGCAATTAAAGAATTATGG |  |
| spaUPF-SEQ | GAGCGTAATACTTACATTTC |  |
| spaDOWNR-SEQ | GCCAATTCCAAATACTGTG |  |
| IM151 | TACATGTCAAGAATAAACTGCCAAAGC |  |
| IM152 | AATACCTGTGACGGAAGATCACTTCG |  |
|  |  |  |
| **Plasmids** |  |  |
| pIMAY | Suicide cloning vector, chloramphenicol resistance | Monk, 2012 |
| pIMAY::Δ*spa* | *spa* deletion vector | This study |

^a^Kindly provided by Dr. J. Harrison, University of Calgary

^b^Collected from subjects enrolled in an ongoing, IRB-approved study (methods)
