# Supplementary material for: Staphylococcus aureus Protein A Mediates Interspecies Interactions at the Cell Surface of Pseudomonas aeruginosa
Source: mBio. 2016 May 24;7(3):e00538-16. doi: 10.1128/mBio.00538-16 (PMC4895107; doi:10.1128/mBio.00538-16)
Supplement: Table S2 — Results of the 4-h biofilm formation assay, measured via crystal violet staining of patient 102 isolates, isolates from 8 additional CF patients, and wild-type and isogenic pslD mutants from the four biofilm matrix usage classes defined by Colvin et al. (2011). (Roman numerals in parentheses next to strain names indicate the matrix usage class for defined strains.) [file mbo003162819st2.docx]

Supplemental Table 2: Four hour biofilm formation as measured by crystal violet assay for Patient 102 isolates, isolates from 8 additional CF patients, and wild type and isogenic *pslD* mutants from the four biofilm matrix usage classes defined by Colvin *et al.,* 2011. Roman numerals in parentheses next to strain name indicate matrix usage class for defined strains.

|  |  | Biofilm Formation (OD595) | | | | | |
| --- | --- | --- | --- | --- | --- | --- | --- |
| Isolate ID | Makes Psl?*** | in LB | (SD) | in *S. aureus* Supernatant | (SD) | T test | Fold Change |
| 102-2 | Y | 0.199 | 0.011 | 0.313 | 0.014 | ** | 1.577 |
| 102-5a | Y | 0.248 | 0.030 | 0.358 | 0.030 | ** | 1.442 |
| 102-5b | Y | 0.257 | 0.025 | 0.315 | 0.012 | * | 1.223 |
| 102-6 | Y | 0.149 | 0.007 | 0.244 | 0.007 | ** | 1.635 |
| 102-7 | Y | 0.205 | 0.006 | 0.277 | 0.018 | ** | 1.349 |
| 102-8 | Y | 0.157 | 0.017 | 0.286 | 0.017 | ** | 1.825 |
| 102-20 | Y | 0.213 | 0.008 | 0.313 | 0.040 | ** | 1.472 |
| 102-21 | N | 0.248 | 0.036 | 0.110 | 0.016 | ** | 0.444 |
| 102-25 | Y | 0.229 | 0.021 | 0.371 | 0.049 | ** | 1.623 |
| 102-26 | Y | 0.268 | 0.025 | 0.315 | 0.030 | * | 1.177 |
| 102-30 | N | 0.307 | 0.053 | 0.092 | 0.004 | ** | 0.300 |
| 102-34 | N | 0.263 | 0.025 | 0.088 | 0.012 | ** | 0.333 |
| 102-35 | N | 0.181 | 0.005 | 0.118 | 0.003 | ** | 0.654 |
| 102-36 | N | 0.243 | 0.025 | 0.124 | 0.017 | ** | 0.511 |
| 102-39 | N | 0.196 | 0.013 | 0.100 | 0.004 | ** | 0.510 |
| 102-40 | N | 0.192 | 0.024 | 0.090 | 0.006 | ** | 0.469 |
| 115-7 | Y | 0.420 | 0.026 | 1.050 | 0.085 | ** | 2.500 |
| 151-10 | Y | 0.424 | 0.054 | 0.823 | 0.128 | ** | 1.942 |
| 159-1 | Y | 0.204 | 0.012 | 0.359 | 0.017 | ** | 1.764 |
| 71-22 | Y | 0.110 | 0.021 | 0.224 | 0.010 | ** | 2.029 |
| 200-4 | N | 0.432 | 0.030 | 0.147 | 0.017 | ** | 0.340 |
| 134-2 | Y | 0.102 | 0.012 | 0.316 | 0.027 | ** | 3.093 |
| 141-2 | N | 0.276 | 0.037 | 0.086 | 0.009 | ** | 0.311 |
| 166-1 | Y | 0.672 | 0.066 | 0.910 | 0.033 | ** | 1.355 |
| PA14 (I) | N | 0.578 | 0.085 | 0.157 | 0.038 | ** | 0.272 |
| Wild type MPAO1 (II) | Y | 1.480 | 0.401 | 2.613 | 0.514 | * | 1.766 |
| MPAO1Δ*pslD* | N | 0.588 | 0.122 | 0.237 | 0.025 | ** | 0.403 |
| Wild type E2 (II) | Y | 1.827 | 0.216 | 2.424 | 0.361 | * | 1.327 |
| E2 Δ*pslD* | N | 0.622 | 0.038 | 0.333 | 0.085 | * | 0.535 |
| Wild type MSH3 (III) | Y | 1.866 | 0.141 | 2.694 | 0.256 | ** | 1.444 |
| MSH3 Δ*pslD* | N | 1.152 | 0.170 | 0.799 | 0.193 | * | 0.693 |
| Wild type CF127 (IV) | Y | 2.814 | 0.264 | 0.288 | 0.007 | * | 0.102 |
| CF127 Δ*pslD* | N | 0.501 | 0.090 | 0.288 | 0.007 | * | 0.574 |
| * p < 0.05 | | | | | | | |
| ** p < 0.001  ***detected by Psl immunoblot | | | | | | | |
