# Supplementary material for: Staphylococcus aureus Protein A Mediates Interspecies Interactions at the Cell Surface of Pseudomonas aeruginosa
Source: mBio. 2016 May 24;7(3):e00538-16. doi: 10.1128/mBio.00538-16 (PMC4895107; doi:10.1128/mBio.00538-16)
Supplement: Table S3 — Results of the 4-h biofilm formation assay, measured via crystal violet staining of a representative P. aeruginosa clinical isolate (102-21), showing that the biofilm inhibition phenotype occurs in supernatant from multiple common lab strains and clinical isolates of S. aureus. (Strains 102-10 and 102-12 are S. aureus clinical isolates that were collected from patient 102, from whom the P. aeruginosa isolate 102-21 was also collected.) [file mbo003162819st3.docx]

Supplemental Table 3: Four hour biofilm formation as measured by crystal violet assay for a representative *P. aeruginosa* clinical isolate (102-21), showing that the biofilm inhibition phenotype occurs in supernatant from multiple common lab strains and clinical isolates of *S. aureus*. SA102-10 and SA102-12 are *S. aureus* clinical isolates that were collected from Patient 102, from whom the *P. aeruginosa* isolate 102-21 was also collected.

|  | 102-21 | | | |
| --- | --- | --- | --- | --- |
| Grown in: | Mean Biofilm (OD595) | SD | T test | Fold Change |
| LB-MOPS | 0.268 | 0.013 | -- | -- |
| SA113 | 0.112 | 0.013 | ** | 0.418 |
| ATCC29213 | 0.148 | 0.014 | ** | 0.552 |
| Newman | 0.13 | 0.009 | ** | 0.486 |
| SA47-34 | 0.143 | 0.006 | ** | 0.533 |
| SA102-10 | 0.175 | 0.02 | ** | 0.653 |
| SA37-12 | 0.15 | 0.018 | ** | 0.559 |
| SA134-5 | 0.121 | 0.014 | ** | 0.45 |
| SA102-12 | 0.122 | 0.015 | ** | 0.457 |
| SA23-71 | 0.145 | 0.028 | ** | 0.541 |
| SA200-3 | 0.105 | 0.008 | ** | 0.394 |
| * p < 0.05 | | | | |
| ** p < 0.001 | | | | |
